# Impact of COVID‐19 on long‐term outcomes in Parkinson's disease

**DOI:** 10.1111/ene.70013

**Published:** 2025-05-07

**Authors:** Roham Hadidchi, Yousef Al‐Ani, Hannah Piskun, Rachel Pakan, Katie S. Duong, Hasan Jamil, Stephen H. Wang, Sonya Henry, Carine W. Maurer, Tim Q. Duong

**Affiliations:** ^1^ Department of Radiology Albert Einstein College of Medicine and Montefiore Medical Center Bronx New York USA; ^2^ Department of Surgery Beth Israel Deaconess Medical Center and Harvard Medical School Boston Massachusetts USA; ^3^ Department of Neurology Stony Brook University Renaissance School of Medicine Stony Brook New York USA; ^4^ Center for Health and Data Innovation Albert Einstein College of Medicine and Montefiore Medical Center Bronx New York USA

**Keywords:** long COVID, Parkinson's disease

## Abstract

**Objectives:**

Patients with pre‐existing Parkinson's disease (PD) face higher risks of severe acute COVID‐19 outcomes than matched controls, but long‐term post‐COVID‐19 outcomes remain largely unknown. This study investigated clinical outcomes up to 3.5 years post‐infection in a Bronx inner‐city PD population.

**Methods:**

This retrospective study evaluated 3512 patients with PD in the Montefiore Health System (January 2016–July 2023), which serves a large diverse population and was an epicenter of the early COVID‐19 pandemic and subsequent infection surges. Comparisons were made with PD patients without a positive SARS‐CoV‐2 test (defined by polymerase chain reaction test). Outcomes were post‐index date all‐cause mortality, major adverse cardiovascular events (MACE), altered mental status, fatigue, dyspnea, headache, psychosis, dementia, depression, anxiety, dysphagia, falls, and orthostatic hypotension. Changes in Levodopa prescriptions were also tabulated. Adjusted hazard ratios (aHR) were computed accounting for competing risks.

**Results:**

PD patients with COVID‐19 had similar demographics but a higher prevalence of pre‐existing comorbidities compared to PD patients without COVID‐19. PD patients with COVID‐19 had greater risk of mortality (aHR = 1.58 [95% CI: 1.03, 2.41]), MACE (aHR = 1.57 [1.19, 2.07]), dyspnea, fatigue, and fall compared to PD patients without COVID‐19. Levodopa dose adjustment was higher post‐infection in the COVID‐19 cohort.

**Conclusions:**

Among PD patients, COVID‐19 was associated with a higher risk of adverse long‐term outcomes. PD patients who survive COVID‐19 may benefit from heightened clinical awareness and close follow‐up. Findings highlight the need to improve post‐COVID care for PD patients to mitigate disease progression and maintain quality of life.

## INTRODUCTION

Patients with pre‐existing Parkinson's disease (PD) have been documented to be at a higher risk of severe acute manifestations of SARS‐CoV‐2 infection compared to matched controls without PD [1]. Acute SARS‐CoV2 infection often triggers acute pulmonary and cardiovascular stress, pathologic hyperinflammation, hypercoagulable state, and secondary infections, which could have long‐lasting effects in some individuals. Indeed, many COVID‐19 survivors reported post‐infection COVID‐19 symptoms [2, 3, 4, 5, 6, 7, 8, 9], developed new COVID‐19‐related disorders [10, 11, 12], and/or experienced worsening of pre‐existing medical conditions [13] months after acute SARS‐CoV2 infection has resolved [14, 15], commonly referred to as long COVID. In individuals with pre‐existing neurological disorders, including PD, SARS‐CoV‐2 infection may provoke accelerated neurodegenerative disease progression resulting in overall worse long‐term outcomes compared to COVID‐19 unexposed individuals. While studies have reported that patients with MS [16, 17, 18, 19, 20, 21, 22, 23, 24, 25, 26] show worsening of disease and long‐term outcomes post‐SARS‐CoV‐2 infection compared to non‐COVID‐matched controls, the potential impact of COVID‐19 on the long‐term outcomes of patients with pre‐existing PD is largely unknown.

The goal of this study was to evaluate the long‐term outcomes of patients with pre‐existing PD up to 3.5 years post‐SARS‐CoV‐2 infection and compare them with controls. Outcomes included all‐cause mortality, major adverse cardiovascular events (MACE), new‐onset dyspnea, fatigue, headache, sleep disturbances, altered mental status, depression, anxiety, dementia, psychosis, fall, orthostatic hypotension, and dysphagia. The study population included patients seen in the Montefiore Health System, which serves a large, diverse population in the Bronx, New York, and its environs, an epicenter of the early COVID‐19 pandemic and subsequent surges of infection.

## METHODS

### Data sources

This retrospective cross‐sectional single‐center study was approved by the Einstein‐Montefiore Institutional Review Board with an exemption for informed consent (#2021‐13658). Data extraction queried records from January 1, 2016, to July 1, 2023, from the Montefiore Health System, which includes multiple hospitals and outpatient clinics in the Bronx. Data were extracted as described previously [27, 28, 29, 30]. The index date was defined as the date of positive polymerase chain reaction (PCR) SARS‐CoV‐2 test for COVID‐19 patients and March 1, 2020, for non‐COVID patients. There were 3512 individuals with pre‐existing PD at index date. The inclusion criteria for the COVID‐19 cohort were as follows: (1) diagnosis of PD, (2) positive PCR SARS‐CoV‐2 test, (3) PD diagnosis occurring prior to positive PCR test, and (4) one or more visits to the Montefiore Health System 14 days or more after their index date. The inclusion criteria for the non‐COVID cohort were as follows: (1) diagnosis with PD prior to March 1, 2020, (2) no positive PCR test, or clinical history of COVID‐19 in the electronic health records (EHR), and (3) one or more visits to the Montefiore Health System 14 days or more after March 1, 2020. Those without a positive test were classified as non‐COVID and the earliest visit after March 1, 2020, was used as index date. A few patients (less than 20) who had a documented positive result from antibody or at‐home SARS‐CoV‐2 test were excluded from both cohorts to limit COVID‐19 patients as being erroneously classified as non‐COVID. PD was defined as physician documentation of “Parkinson's disease” (Systematized Nomenclature of Medicine Clinical Terms 49049000). Additionally, patients with a documented history of atypical Parkinsonian disorders were excluded (40 patients with vascular, postencephalitic, or drug‐induced parkinsonism). Studies using an earlier version of this large database to address different questions have been reported [10, 11, 12, 13, 29, 30, 31, 32, 33, 34, 35, 36, 37, 38, 39, 40].

### Variables

Demographic data included age at index date, sex, race, and ethnicity. Pre‐existing comorbidities included hypertension (HTN), type 2 diabetes (T2D), chronic obstructive pulmonary disease (COPD), asthma, congestive heart failure (CHF), chronic kidney disease (CKD), coronary artery disease (CAD), and obesity (defined as a body mass index of >30 or a diagnosis of obesity) that were diagnosed at or prior to the index date. Tobacco use status was defined as the patient self‐reporting tobacco use at any point in their lifetime. Pre‐existing sleep disturbance, headaches, depression, anxiety, dementia, psychosis, orthostatic hypotension, and dysphagia were also tabulated. Data about corticosteroid (hydrocortisone, methylprednisolone, dexamethasone, and prednisone) and antiviral drug (ritonavir and remdesivir) prescriptions for the treatment of COVID‐19, and critical illness status (requiring intensive care unit or invasive mechanical ventilation) were collected.

### Outcomes

The outcomes included all‐cause mortality 14 days or later after index date and major adverse cardiovascular events (MACE), defined as the composite of stroke, myocardial infarction, new‐onset heart failure, and cardiogenic shock 30 days or more after index date. The secondary outcomes included development of the following conditions or symptoms 30 days or more post‐index date: (i) dyspnea, (ii) fatigue (patient‐reported symptom), (iii) sleep disturbances (insomnia, irregular sleep–wake pattern, central or obstructive sleep apnea, phase regression and advancement syndromes, rapid eye movement sleep behavior disorder, or restless leg syndrome), (iv) altered mental status (due to any cause), (v) headache (non‐migrainous), (vi) depression (major depressive disorder diagnosis, PHQ‐9 score of 10 or higher), and (vii) anxiety (organic or generalized anxiety disorder diagnosis, GAD‐7 score of 10 or higher, GAD‐2 score of 3 or higher). PD‐related outcomes included: (i) dementia (newly documented), (ii) psychosis (newly documented), (iii) fall, (iv) orthostatic hypotension, and (v) dysphagia. Patients who at index date had a history of fatigue, anxiety, depression, sleep disturbances, headaches, dementia, psychosis, orthostatic hypotension, and dysphagia were excluded from the analysis of the respective outcome.

To assess the impact of SARS‐CoV‐2 infection on PD medications, a history of PD medication prescriptions and their daily dosages were collected. PD medications included carbidopa, levodopa, entacapone, opicapone, selegiline, rasagiline, apomorphine, pramipexole, ropinirole, rotigotine, and amantadine. Using the daily dose and the active agent, the levodopa‐equivalent dose (LED) of each prescription was calculated [41].

### Analysis and statistics

Python version 3.10.12 and the lifelines package, the survival, survminer, and cmprsk packages in RStudio version 4.3.2 (RStudio, PBC, Boston, MA), and GraphPad Prism 9 version 10.1.1 (GraphPad Software, Boston, Massachusetts, USA) were used for data processing and statistical analyses. Group comparison of categorical variables used, chi‐square test, and group comparison of continuous variables used the independent *t*‐test. Kaplan–Meier curves and log‐rank analysis were performed for all‐cause mortality. Cumulative incidence function and Fine‐Gray sub‐distribution hazards model analysis were performed for post‐index data for all other secondary outcomes associated with SARS‐CoV‐2 infection. In the univariate analysis, hazard ratios (HR) were computed using the Fine‐Gray model to account for mortality as a competing risk for developing the outcomes. In addition, to adjust for other covariates, the multivariate Cox proportional hazards model was used to calculate adjusted hazard ratios (aHR) and 95% confidence interval (CI) for outcomes. The covariates adjusted for included SARS‐CoV‐2 infection status, all comorbidities, age, sex, race, and ethnicity, as they are commonly associated with health outcomes and disease progression in those with PD [42, 43, 44, 45]. Age at onset of PD and other disease features that could not be obtained were not included.

To analyze the impact of SARS‐CoV‐2 infection on the dosage of levodopa equivalent dose PD medications the Generalized Estimating Equations (GEE) model was used. Levodopa equivalent dose change is that of exponentiated coefficient of GEE model. The analysis considered several predictors, including the elapsed time since index date. COVID‐19 status was treated as a binary variable, where patient observations shifted from 0 (before infection) to 1 (after infection) to assess immediate changes in medication doses. Additionally, an interaction term between the time from the baseline and COVID‐19 status was included to examine long‐term trend. Outcomes were adjusted for age, race, and ethnicity. The analysis was performed for male and female patients separately. Given the skewed distribution of the medication dose data, a log transformation was applied to normalize the distribution, with a constant of 1 added to accommodate medications with zero LED, particularly for patients on entacapone and opicapone, which do not directly contribute to the LED when prescribed without levodopa. The analysis utilized a Gaussian distribution assumption within the GEE framework to model the continuous outcome of LED. The choice of an autoregressive order 1 correlation structure was made to account for the correlation of repeated measures within the same patient over time.

*p*‐values <0.05 were considered statistically significant.

## RESULTS

### Demographics

**FIGURE 1:**
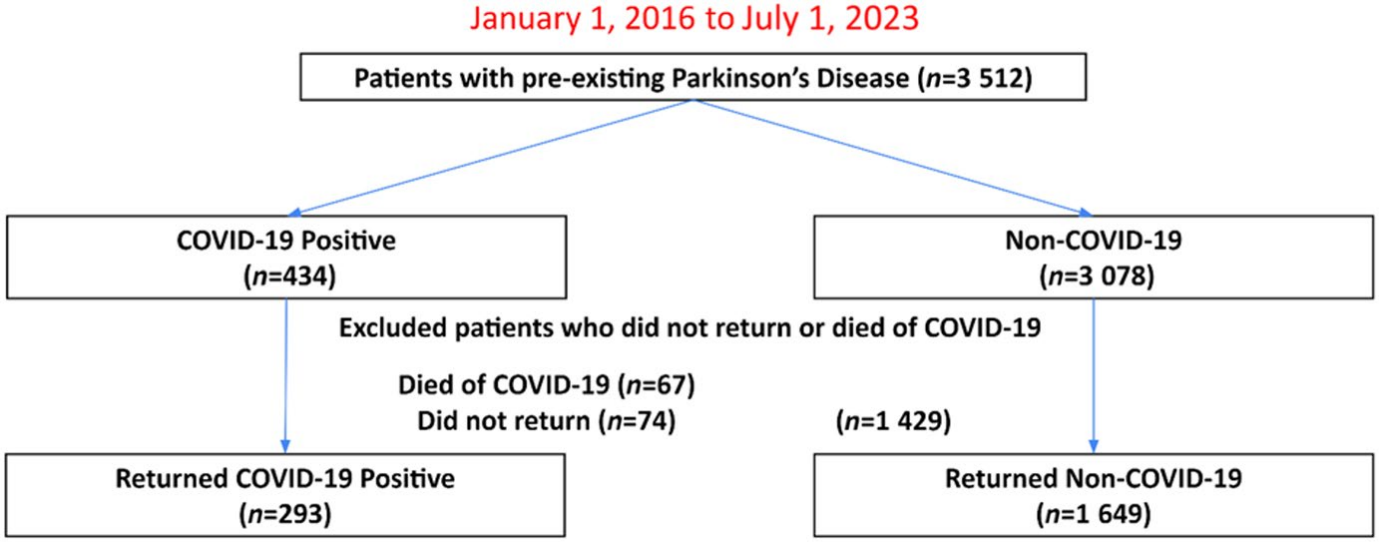
Flowchart of patient selection.

Among the 3512 patients with pre‐existing PD in the Montefiore Health System from Jan 1, 2016, to July 1, 2023, 434 (14%) had a documented positive SARS‐CoV‐2 PCR test and 3078 did not (Figure 1). Sixty‐seven patients with COVID‐19 (15.43%) died within the first 2 weeks of a COVID‐19 positive test. Excluding those who died and those who did not return to our health system, the final cohort used to assess long‐term outcomes included 293 COVID‐19 (17.7%) and 1649 non‐COVID‐19 patients with pre‐existing PD.

PD patients with and without COVID‐19 did not differ in terms of age or sex. PD patients with COVID‐19 were less likely to be White and more likely to be Hispanic. PD patients with COVID‐19 had a higher prevalence of all major pre‐existing comorbidities (*p* < 0.05 for all) except tobacco use (*p* = 0.2). PD patients with COVID‐19 also had a higher prevalence of pre‐existing headache, depression, anxiety, dementia, and psychosis (*p <* 0.05 for all; Table 1). Of the PD patients with COVID‐19, 28.67% were treated with steroids, 29.01% were treated with antiviral drugs, and 2.05% had critical illness.

**TABLE 1:** Demographics and comorbidities of COVID‐19 and non‐COVID‐19 patients with pre‐existing Parkinson's disease (*n* = 1942). Bold values indicate statistical significance *p* < 0.05.

	COVID+ (*n* = 293)	COVID− (*n* = 1649)	*p* values
Age at Index Date, mean ± SD (years)	75.54 ± 11.41	74.64 ± 11.48	0.22
Follow‐up duration, mean ± SD (years)	1.73 ± 1.05	3.18 ± 0.59	
Female, *n* (%)	152 (51.88)	819 (49.67)	0.53
Race and ethnicity, *n* (%)			
White	60 (20.48)	437 (26.50)	**0.035**
Black	76 (25.94)	359 (21.77)	0.13
Asian	9 (3.07)	47 (2.85)	0.98
Other Races	148 (50.51)	806 (48.88)	0.65
Hispanic	130 (44.37)	596 (36.14)	**0.0089**
Pre‐existing comorbidities, *n* (%)			
Hypertension	247 (84.30)	1194 (72.41)	**<0.0001**
Type‐2 diabetes	172 (58.70)	762 (46.21)	**0.0001**
COPD	51 (17.41)	156 (9.46)	**<0.0001**
Asthma	69 (23.55)	227 (13.77)	**<0.0001**
Congestive heart failure	82 (27.99)	244 (14.80)	**<0.0001**
Chronic kidney disease	114 (38.91)	426 (25.83)	**<0.0001**
Coronary artery disease	117 (39.93)	362 (21.95)	**<0.0001**
Current or past tobacco use	113 (38.57)	569 (34.51)	0.2022
Obesity	82 (27.99)	342 (20.74)	**0.0071**
Pre‐existing symptoms and disorders, *n* (%)			
Sleep disturbances	71 (24.23)	363 (22.01)	0.45
Headache	16 (5.46)	57 (3.45)	0.14
Depression	100 (34.13)	306 (18.56)	**<0.0001**
Anxiety	82 (27.99)	323 (19.59)	**0.0015**
Dementia	143 (48.81)	573 (34.75)	**<0.0001**
Psychosis	50 (17.06)	194 (11.76)	**0.015**
Orthostatic hypotension	23 (7.85)	94 (5.70)	0.120
Dysphagia	4 (1.37)	6 (0.36)	0.078
Acute COVID‐19 treatments, *n* (%)			
Steroids	84 (28.67)	N/A	N/A
Antiviral drugs	85 (29.01)	N/A	N/A
Critical illness	6 (2.05)	N/A	N/A

### Outcomes

**FIGURE 2:**
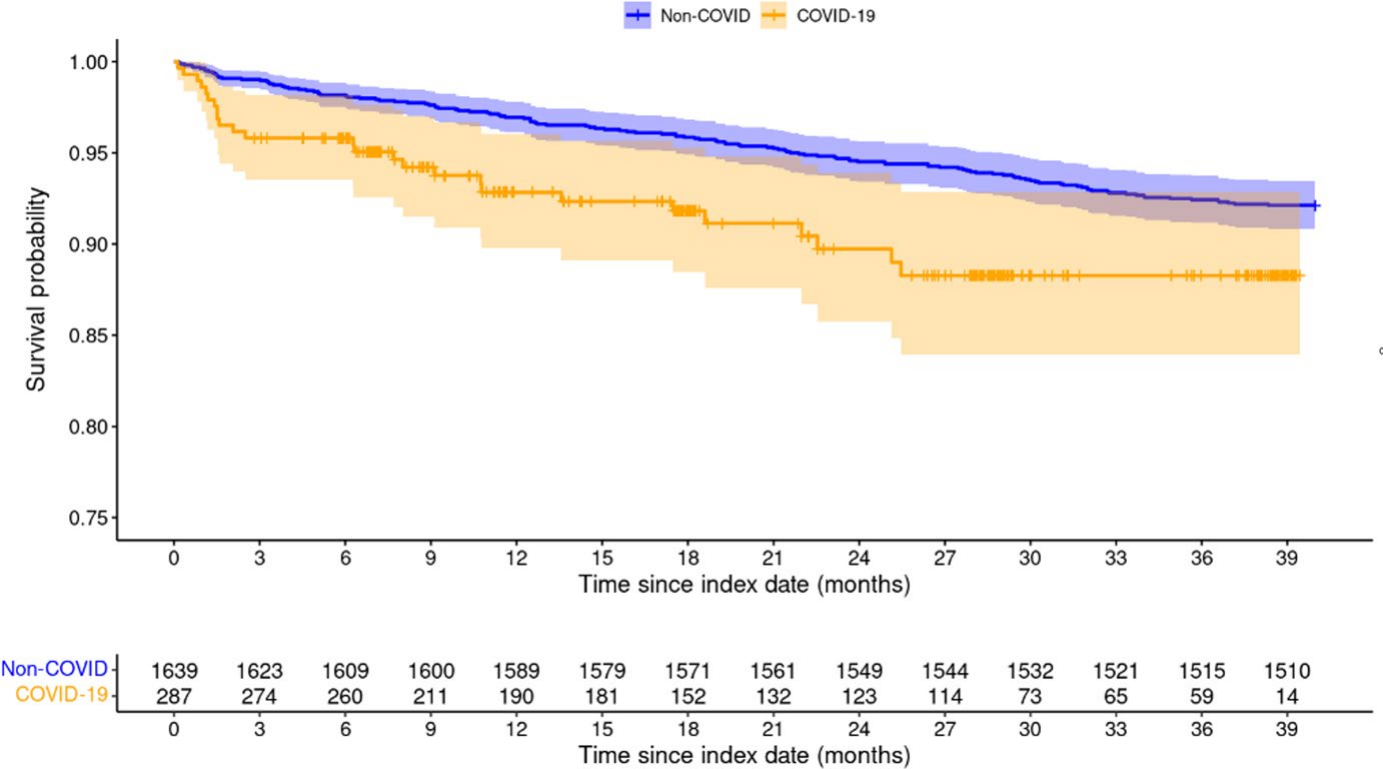
Kaplan–Meier curve of all‐cause mortality 14 days or more post‐index date between COVID‐19 and non‐COVID patients with PD.

COVID‐19 patients with PD had significantly higher all‐cause mortality compared to non‐COVID patients with PD (unadjusted log‐rank HR = 2.06 [CI:1.39, 3.04] *p* = 0.0002; Figure 2). Notably, there was a comparatively higher risk of mortality within the first 3 months in the COVID‐19 cohort.

SARS‐CoV‐2 infection was significantly associated with increased long‐term risk of mortality (aHR = 1.58 [95% CI:1.03, 2.41] *p* = 0.03), along with age (aHR = 1.02 [1.00, 1.04] *p* = 0.01), CHF (aHR = 1.58 [1.07, 2.37] *p* = 0.02), and COPD (aHR = 1.80 [1.17, 2.77] *p* = 0.01). Other race versus White was associated with a lower risk of mortality (aHR = 0.60 [0.38, 0.94] *p* = 0.03; Table 2).

**TABLE 2:** Cox proportional hazard ratios for (HR) for all‐cause mortality. Bold values indicate statistical significance *p* < 0.05.

Covariates	Hazard ratio	95% [CI]	*p* value
Age (age)	1.02	1.00–1.04	**0.01**
SARS‐CoV‐2 infection	1.58	1.03–2.41	**0.03**
Female versus male	0.74	0.53–1.03	0.08
Black versus White	0.84	0.55–1.28	0.41
Asian versus White	1.25	0.58–2.70	0.57
Other race versus White	0.60	0.38–0.94	**0.03**
Hispanic versus non‐Hispanic	0.84	0.53–1.33	0.47
Hypertension	1.09	0.69–1.72	0.72
Type 2 diabetes	1.27	0.88–1.82	0.20
Congestive heart failure	1.58	1.07–2.31	**0.02**
Chronic obstructive pulmonary disease	1.80	1.17–2.77	**0.01**
Asthma	1.00	0.64–1.55	0.99
Coronary artery disease	1.18	0.82–1.70	0.36
Chronic kidney disease	1.40	0.97–2.00	0.07
Current or past tobacco use	0.94	0.67–1.32	0.74
Obesity	1.13	0.77–1.66	0.53

**FIGURE 3:**
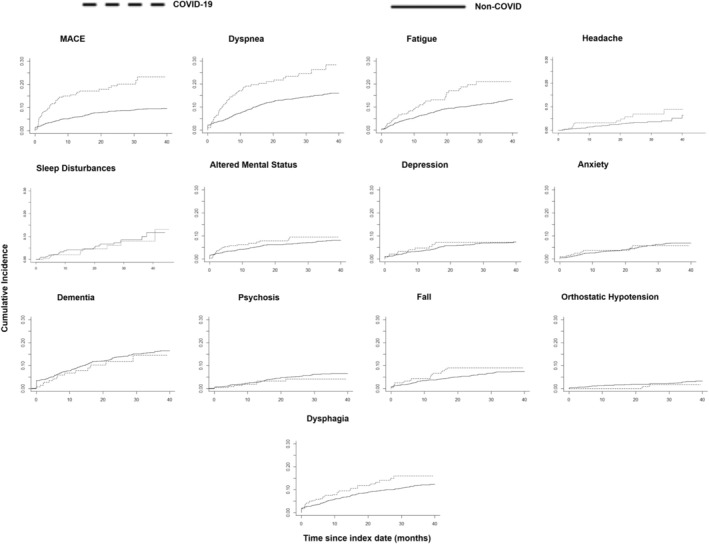
Cumulative incidence curves of outcomes (post‐index date in months). MACE, major adverse cardiovascular events.

Figure 3 shows the cumulative incidences for MACE, dyspnea, fatigue, sleep disturbances, altered mental status, headache, depression, and anxiety, as well as dementia, psychosis, fall, orthostatic hypotension, and dysphagia. Table 3 shows the results of univariate Fine‐Gray HR and multivariate Cox proportional HR for the outcomes associated with SARS‐CoV‐2 infection. With the univariate analysis, SARS‐CoV‐2 infection was significantly associated with an increased long‐term risk of MACE, dyspnea, and fatigue. With the multivariate analysis, SARS‐CoV‐2 infection was significantly associated with an increased long‐term risk of MACE (aHR = 1.57 [95% CI:1.19, 2.07], *p* < 0.005), dyspnea (aHR = 1.44 [1.11, 1.87], *p* < 0.01), fatigue (aHR = 1.49 [1.12, 1.97] *p* < 0.01), and fall (aHR = 1.39 [1.01, 1.92] *p* = 0.04).

**TABLE 3:** Univariate Fine‐Gray HR and multivariate Cox proportional HR of post‐index date outcomes.

Outcomes	Univariate			Multivariate		
	HR	95% [CI]	*p*‐value	HR	95% [CI]	*p*‐value
MACE	2.22	1.61–3.05	**<0.0001**	1.72	1.31–2.26	**<0.005**
Dyspnea	1.90	1.42–2.53	**<0.0001**	1.44	1.11–1.87	**0.01**
Fatigue	1.65	1.17–2.33	**0.004**	1.49	1.12–1.97	**0.01**
Sleep disturbances	0.81	0.39–1.67	0.56	0.76	0.36–1.59	0.46
Altered mental status	1.29	0.82–2.03	0.27	1.29	0.90–1.77	0.17
Headache	1.44	0.48–4.35	0.52	1.33	0.43–4.09	0.62
Depression	1.08	0.82–1.43	0.57	1.35	0.89–2.04	0.16
Anxiety	1.11	0.82–151	0.49	1.28	0.85–1.93	0.24
Dementia	0.82	0.48–1.41	0.47	1.03	0.65–1.63	0.89
Psychosis	0.68	0.31–1.47	0.33	0.93	0.59–1.45	0.75
Fall	1.37	0.85–2.21	0.19	1.39	1.01–1.92	**0.04**
Orthostatic hypotension	0.39	0.09–1.65	0.20	1.25	0.83–1.87	0.28
Dysphagia	1.37	0.94–1.98	0.10	1.47	0.97–2.23	0.07

*Note*: Hazard ratios (HR) show the relative risk of having the respective outcome if infected with SARS‐CoV‐2. Multivariate analysis adjusted for other covariates: All major comorbidities, age, sex, race, and ethnicity. Bold values indicate statistical significance *p* < 0.05.

Table 4 shows that the GEE analysis on the impact of COVID‐19 on LED adjustments revealed a notable increase in LED for both genders post‐infection. COVID‐19 status was significantly associated with LED adjustments in both male and female. Males exhibited a slight but statistically significant decrease in medication dosage over time post‐infection (0.22% per month since the index date, *p* = 0.01), whereas females showed a consistent medication dosage level over the observed period but not statistically significant (*p* = 0.842). Levodopa equivalent dose was not associated with race, ethnicity, or age.

**TABLE 4:** Associated changes in levodopa equivalent dose after SARS‐CoV‐2 infection for those who had pre‐ and post‐index date prescription recorded. Bold values indicate statistical significance *p* < 0.05.

Variable	Males (*n* = 629)			Females (*n* = 633)		
	LED change	[95% CI]	*p*‐value	LED change	[95% CI]	*p*‐value
SARS‐CoV‐2 infection	1.230	1.0100–1.4978	**0.03**	1.2473	1.0013–1.5537	**0.04**
Time Since Index Date (months)	1.000	1.0000–1.0001	0.28	1.0001	1.0000–1.0002	**0.01**
Time Since Index Date × SARS‐CoV‐2 Infection	0.9978	0.9961–0.9996	**0.01**	1.0000	0.9995–1.0004	0.84
Asian versus White	0.8566	0.5187–1.4146	0.54	0.9044	0.519–1.5762	0.72
Black versus White	0.9721	0.7961–1.1870	0.78	0.9965	0.8223–1.2075	0.97
Hispanic versus non‐Hispanic	0.9308	0.7854–1.1033	0.40	0.9990	0.8466–1.1788	0.99
Other race versus White	1.0369	0.8745–1.2295	0.67	1.2080	0.9866–1.4792	0.06
Age	0.9962	0.9901–1.0022	0.21	0.9995	0.9931–1.0059	0.87

## DISCUSSION

This study investigated the clinical outcomes of patients with pre‐existing PD up to three and a half years post‐SARS‐CoV‐2 infection in a large, racially and ethnically diverse population in the Bronx, New York. The major findings are that SARS‐CoV‐2 infection was significantly associated with higher risk for mortality, MACE, dyspnea, fatigue, and fall in PD patients. LED adjustment was higher post‐infection in the male COVID‐19 patients PD compared to non‐COVID‐19 patients with PD.

About 12% of PD patients had a positive COVID‐19 test. A meta‐analysis in 2021 estimated the average SARS‐CoV‐2 infection rate to be approximately 4%. SARS‐CoV‐2 infection rates are highly dependent on region, COVID‐19 testing rate, duration over which the study included, and the populations being studied [46]. The Bronx was hit hard by the early pandemic and subsequent waves of infection and minority populations have been reported to be at a high risk of infection [28]. The high prevalence of major comorbidities and neuropsychiatric conditions among the PD patient cohort could further contribute to the comparatively high infection rate.

PD patients with COVID‐19 showed increased mortality risk, most prominent in the first 3 months post‐infection, suggesting such risk was likely COVID‐19 related. The adjusted hazard ratio for long‐term risk for mortality of PD patients with SARS‐CoV‐2 infection over the study period was 1.58 times higher than PD patients without COVID‐19. This risk was comparable to risk posed by pre‐existing COPD (aHR = 1.80 [95% CI: 1.17, 2.77]) and CHF (aHR = 1.58 [95% CI: 1.07, 2.31]), underscoring the contribution of SARS‐CoV‐2 infection to long‐term mortality. Age was also a significant risk factor as expected with a 2% increased risk for every year of age.

PD patients who survived acute SARS‐CoV‐2 infection also had a higher adjusted long‐term risk of developing MACE compared to controls. COVID‐19 patients have previously been shown to have an increased incidence of long‐term MACE [47], possibly secondary to the impact of infection on acute pulmonary and cardiovascular stress [48, 49]. SARS‐CoV‐2 infection was significantly associated with higher adjusted risk for dyspnea, fatigue, and fall in PD patients up to three and a half years post‐pandemic. Although many of these symptoms occur in PD patients, they are not exclusive to those with PD [50, 51, 52, 53, 54]. Our cohort consisted of a large proportion of minorities. Notably, other races (non‐White, non‐Black, and non‐Asian) were at lower risk of long‐term mortality than Whites. These health disparity data need to be interpreted with caution because our cohort consisted of a relatively small proportion of White patients.

Similar studies of longer‐term outcomes of PD patients with COVID‐19 are sparse. None thus far have directly compared the long‐term outcomes of PD patients, with and without a history of COVID‐19. Bougea et al. [55] studied PD patients with COVID‐19 at baseline and at 6 months post‐infection using clinicodemographic questionnaire. They found that among those who reported post‐COVID‐19 syndrome, levodopa equivalent daily dose and Unified Parkinson's Disease Rating Scale Part III increased after infection, indicating possibly elevated disease activity. Weiss et al. [56] analyzed data from over 2000 individuals with PD 6 months to 2 years post‐COVID‐19 in Germany. Their analysis revealed that at follow‐up, depression and fatigue were associated with difficulties with daily activities, perceived health‐related quality of life, chronic exhaustion, unrestful sleep, and impaired concentration. Brown et al. [57] demonstrated that during the first 2 months of the pandemic, PD patients with COVID‐19 experienced higher incidence of worsening and new occurrence of a variety of PD symptoms compared to non‐COVID PD patients.

### Mechanisms

Elevated long‐term adverse outcomes among COVID‐19 patients could be the result of an acute systemic inflammatory response [58]. SARS‐CoV‐2 could trigger cytokine storm, interleukin, and tumor necrosis factor release [59]. SARS‐CoV‐2 may induce neuroinflammation and dopaminergic degeneration by triggering proinflammatory cytokines in microglia [58]. Persistent chronic inflammation and neuronal damage post‐SARS‐CoV‐2 infection could contribute to worse long‐term outcomes. Biomarkers for central nervous system injury in cerebrospinal fluid have been found to be elevated in patients with COVID‐19 and associated with neurological symptoms and disease severity [60]. These markers included neurofilament light chain protein, tau, and glial fibrillary acidic protein [60]. SARS‐CoV‐2 infection could exacerbate oxidative stress, which has been shown to induce damage dopaminergic neurons in the substantia nigra in animal models [61]. Notably, Wang et al. [62] report that the spike protein interacts with α‐synuclein and promotes α‐synuclein aggregation. The S1 domain could induce mitochondrial dysfunction, oxidative stress, and cytotoxicity. Thus, the S1 domain of SARS‐CoV‐2 could promote the aggregation of α‐synuclein in the cellular model of synucleinopathy and may contribute to the pathogenesis of PD.

### Social impact of pandemic on PD outcomes

We did not find SARS‐CoV‐2 infection to be associated with risk of new‐onset depression or anxiety, but the social impact of the pandemic, such as the effects of isolation, psychological stress, reduced physical activity, unhealthy diet, and reduced access to care, could also increase stress and exacerbate PD symptoms, resulting in overall worse long‐term outcomes [63, 64]. Patients with PD exhibit cognitive and motor inflexibility, are more likely to have anxiety and depression and are more susceptible to the effects of psychological stress [65, 66]. Increased stress levels during the COVID‐19 pandemic could worsen various motor symptoms and reduce the efficacy of dopaminergic medication [64, 67]. Rezayi et al. [68] found negative effects of the COVID‐19 pandemic on health‐related quality of life and its determinants in patients with PD and their caregivers. Kataoka et al. [69] found that during the early COVID‐19 pandemic, there was an increase in the severity of depression in patients with PD. Kinger et al. [70] conducted a survey study of PD patients before and during the COVID‐19 pandemic and found a significant increase in apathy, but not in depression or anxiety, during the pandemic. Anxiety and depression, but not apathy, were correlated with the impact of COVID‐19. The COVID‐19 pandemic also resulted in worsening of sleep, cognitive disturbances, autonomic dysfunction, anxiety, depression, appetite disorders, repeated falls, and pain in PD patients [57]. We found psychiatric symptoms (depression, anxiety, and psychosis) were not significantly different between COVID‐19 and non‐COVID‐19 patients with PD 3.5 years post‐infection, and these findings of suggest that these symptoms might be in part a result of social impact of COVID‐19, more than the virus itself.

### Strengths and limitations

The strength of this study included a long follow‐up time of 3.5 years post‐infection, a large sample size, and a diverse and underserved population. This study has several limitations. Our findings were limited to patients who returned to our health system. Although patient records included those who returned for any medical reason, including but not limited to routine office visits, patients who came to our health system could likely have had more severe COVID‐19. There could be errors in EHR. The uneven distribution between COVID‐19 and non‐COVID‐19 groups could result in asymmetry between sensitivity and specificity, as well as unusually large or small odds ratio and asymmetry in the 95% confidence intervals. Outcomes might be affected by other factors including COVID‐19 vaccinations, strain of SARS‐CoV‐2, and disease severity. Vaccine status was not reliably recorded if patients received vaccines outside of the Montefiore Health System, and thus was not included in our analysis. Disease features such as PD stage, age of PD onset, and severity of motor and non‐motor symptoms could not be reliably extracted or available in our EHR, and therefore were not analyzed. There could be other unintended patient selection biases and latent confounding.

## CONCLUSION

This study assessed the long‐term outcomes of patients with PD up to three and a half years post SARS‐CoV‐2 infection in an underserved urban population. PD patients with COVID‐19 had a greater adjusted risk of long‐term mortality, greater adjusted risk of developing a major adverse cardiovascular event, dyspnea, fatigue, and fall compared to PD patients without COVID‐19. Patients with PD who survive COVID‐19 may benefit from additional clinical follow‐up.

## AUTHOR CONTRIBUTIONS

**Roham Hadidchi:** Conceptualization; investigation; formal analysis; validation; writing – review and editing; writing – original draft. **Yousef Al‐Ani:** Conceptualization; writing – original draft; formal analysis. **Hannah Piskun:** Writing – original draft; writing – review and editing. **Rachel Pakan:** Investigation; formal analysis; writing – original draft. **Katie S. Duong:** Writing – review and editing; writing – original draft; validation. **Hasan Jamil:** Formal analysis; investigation; validation; writing – original draft. **Stephen H. Wang:** Validation; writing – review and editing; methodology; conceptualization. **Sonya Henry:** Data curation; methodology. **Carine W. Maurer:** Methodology; conceptualization; writing – review and editing; validation. **Tim Q. Duong:** Conceptualization; writing – original draft; writing – review and editing; validation; methodology; supervision.

## CONFLICT OF INTEREST STATEMENT

All authors declare no conflict of interest.

## Data Availability

The data that support the findings of this study are available from the corresponding author upon reasonable request.
